# Investigating the feasibility, acceptability and appropriateness of outreach case management in an urban Aboriginal and Torres Strait Islander primary health care service: a mixed methods exploratory study

**DOI:** 10.1186/s12913-016-1428-0

**Published:** 2016-05-13

**Authors:** Deborah A. Askew, Samantha J. Togni, Philip J. Schluter, Lynne Rogers, Sonya Egert, Nichola Potter, Noel E. Hayman, Alan Cass, Alex D. H. Brown

**Affiliations:** Southern Queensland Centre of Excellence in Aboriginal and Torres Strait Islander Primary Health Care, Queensland Health, Wirraway Pde, Inala Queensland 4077 Australia; The University of Queensland, Discipline of General Practice, Brisbane, Australia; Baker IDI Heart and Diabetes Institute, Alice Springs, Australia; Menzies School of Health Research, Darwin, Australia; University of Canterbury, School of Health Sciences, Christchurch, New Zealand; The University of Queensland, School of Nursing, Midwifery and Social Work, Brisbane, Australia; South Australian Health and Medical Research Institute, North Terrace Adelaide, Australia

**Keywords:** Aboriginal and torres Strait Islander peoples’ health, Chronic disease care, Coordinated care, Case management, Primary health care

## Abstract

**Background:**

The disparities in health and life expectancy of Aboriginal and Torres Strait Islander peoples compared to non-Indigenous Australians are well documented. Chronic diseases are a leading contributor to these disparities. We aimed to determine the feasibility, acceptability and appropriateness of a case management approach to chronic disease care integrated within an urban Aboriginal and Torres Strait Islander primary health care service.

**Methods:**

The *H*ome-based, *O*utreach case *M*anagement of chronic disease *E*xploratory (HOME) Study provided holistic, patient centred multidisciplinary care for Aboriginal and Torres Strait Islander people with chronic disease. A developmental evaluation approach supported the implementation and ongoing adaptations in the delivery of the model of care, and ensured its alignment with Aboriginal and Torres Strait Islander peoples’ understandings of, and approaches to, health and wellbeing. In-depth, semi-structured interviews were conducted with nine patient participants (one interview also included a participant’s spouse) and 15 health service staff and key themes were identified through an iterative reflective process. Quantitative data were collected directly from patient participants and from their medical records at baseline, 3 and 6 months. Patient participants’ baseline characteristics were described using frequencies and percentages. Attrition and patterns of missing values over time were evaluated using binomial generalized estimating equation (GEE) models and mean differences in key clinical outcomes were determined using normal GEE models.

**Results:**

Forty-one patients were recruited and nine withdrew over the 6 month period. There was no evidence of differential attrition. All participants (patients and health service staff) were very positive about the model of care. Patient participants became more involved in their health care, depression rates significantly decreased (*p* = 0.03), and significant improvements in systolic blood pressure (*p* < 0.001) and diabetes control (*p* = 0.05) were achieved.

**Conclusions:**

The exploratory nature of our study preclude any definitive statements about the effectiveness of our model of care. However, staff and patients' high levels of satisfaction and improvements in the health and wellbeing of patients are promising and suggest its feasibility, acceptability and appropriateness. Further research is required to determine its efficacy, effectiveness and cost-effectiveness in improving the quality of life and quality of care for Aboriginal and Torres Strait Islander peoples living with chronic disease.

## Background

The disparities in health and life expectancy of Aboriginal and Torres Strait Islander peoples compared to non-Indigenous Australians are well documented [1], with the life-expectancy gap being evidence of one of contemporary Australia’s most enduring equity, equality and social justice divides [2]. Chronic diseases (CDs) are a leading contributor to these disparities, in both relative and absolute terms [3], and although the mortality gap due to respiratory and circulatory diseases has narrowed, this gap has widened when diabetes, cancer and kidney disease are considered [4]. Additionally, morbidity and mortality due to these CDs remain significantly higher for Aboriginal and Torres Strait Islander peoples than their non-Indigenous counterparts [4]. Despite well-meaning intentions of governments, researchers and service providers, dating from the introduction of the Aboriginal Protection boards in the late 19^th^ Century to the current Australian Government’s Indigenous Australians’ Health Program, these disparities remain [5, 6]. Why?

The health inequalities experienced by Australia’s Aboriginal and Torres Strait Islander peoples compared to non-Indigenous Australians date from the time of white settlement and have been perpetuated by the continuing effects of colonisation, intergenerational trauma and widespread social and economic disadvantage [2, 5]. Aboriginal and Torres Strait Islander people suffer from reduced economic and education opportunities, limited physical infrastructure and poorer social conditions which further contributes to their inequitable health status [5–7]. Consistent with the social determinants of health viewpoint, Aboriginal and Torres Strait Islander peoples have a holistic and collective understanding of health that encompasses the social, emotional and cultural wellbeing of the whole community. An individual’s health status is inextricably linked to the health status of their whole community. Thus, reductionist compartmentalised and individualistic biomedical approaches to addressing health disparities often have significant limitations for Aboriginal and Torres Strait Islander peoples because these approaches are largely antithetical to their holistic conceptualisations of health.

To mitigate these limitations, alternative approaches are needed that are informed by the holistic and collective understanding of health of Aboriginal and Torres Strait Islander peoples. Models of care are needed that simultaneously deliver evidence based, best practice care and privilege Aboriginal and Torres Strait Islander peoples’ understanding of health and health care needs. The majority of CD care occurs in the primary health care setting, and therefore effective models of CD care need to be integrated with this sector [8]. Primary health care based outreach case management is perhaps one such approach that can exploit the strengths of biomedical science in a culturally appropriate manner.

Outreach case management is a collaborative process of care coordination that facilitates intensive multidisciplinary care for individuals in their home or other settings away from traditional health care facilities [9]. Although no universally accepted definition of case management exists, there is general agreement that it is comprised of six core functions, namely: assessment, planning, linking, monitoring, advocacy and outreach [10]. Case management has been demonstrated to be effective in improving clinical indicators, quality of life and functionality, patient satisfaction, adherence to treatment, self care and service use [11]. Inherent to case management is a holistic approach to health care which recognises the interconnectedness of psychosocial factors and physical and mental health. This conception and operationalization of health care appears more closely aligned to Aboriginal and Torres Strait Islander peoples understanding of health than many other conventional approaches.

Despite the high burden of CDs among Aboriginal and Torres Strait Islander peoples, few intervention trials have sought to implement and evaluate novel approaches to reducing this disparity. Patient-centred, home-based, outreach models of CD management that are informed by Aboriginal and Torres Strait Islander conceptualisations of health have the potential to improve the biomedical and psychosocial health status for Aboriginal and Torres Strait Islander people with CD. Therefore, we developed and implemented such a programme in an urban Aboriginal and Torres Strait Islander primary health care service. In this paper, we describe the model of care and report on its feasibility, acceptability and appropriateness to Aboriginal and Torres Strait Islander people with CD and their primary health care service after the first 6 months of its implementation.

## Methods

### Aim and objectives of the HOME Study

The *H*ome-based, *O*utreach case *M*anagement of chronic disease *E*xploratory (HOME) Study aimed to implement a home-based, case management model of patient centred multidisciplinary care for Aboriginal and Torres Strait Islander people with complex CD that was integrated into a primary health care service. The initial evaluation, reported here, aimed to determine the feasibility, acceptability and appropriateness of this model of care. Further evaluation will explore the impact of the model of care on patient participants’ bio-psychosocial health at 12 months; how a primary health care service incorporates this model of care into its usual practice; identification of the key elements of this model of care and how they differ from usual chronic disease management in the primary health care service; and assessment of how participants’ social contexts affect their health, wellbeing and CD management.

### Setting

The HOME Study was conducted at the Southern Queensland Centre of Excellence in Aboriginal and Torres Strait Islander Primary Health Care (COE), a Queensland Government general practice located in Inala, a south-western suburb of Brisbane that provides primary health care predominantly to Aboriginal and Torres Strait Islander people [12]. The HOME Study employed two case managers (CMs), both registered nurses, and the Study team also included the COE Research Director, a Program Coordinator, an Indigenous Research Officer (IRO) and the evaluator. One CM (non-Aboriginal) had previously worked in a similar role in the United Kingdom, and the other (an Aboriginal woman) brought her Aboriginal health worker and community nurse background to the team, in addition to her cultural and community knowledge. The IRO, a member of the local Aboriginal and Torres Strait Islander community, brought in-depth knowledge, understanding and connectivity with this community. The evaluator had considerable experience in developing and implementing applied research and evaluation projects within Indigenous primary health care settings, in addition to experience in organisational development. The Research Director had experience in conducting research in the Inala Aboriginal and Torres Strait Islander community [13]. An advisory group consisting of the Investigators, the evaluator, and the COE Clinical Director, Nurse Unit Manager (NUM), and Research Director provided research governance and oversight.

### Participants

To be eligible to participate in the HOME Study, patients had to:

1. be a regular attendee of the health service (operationally defined for this project as having attended the health service at least twice per year over the last 2 years);
2. be able to provide informed consent;
3. have a confirmed diagnosis of at least one of the following CDs: Type 2 Diabetes (T2D), cardiovascular disease (CVD) (including congestive cardiac failure, history of coronary artery disease or stroke), chronic respiratory disease, or chronic kidney disease (eGFR between 15 and 60) (CKD);
4. be perceived by the CoE NUM as being likely to benefit from care coordination by a CM;
5. live within a geographically accessible area for regular in-home follow-up (operationally defined for this project as being within an one hour drive from the health service); and
6. be of Aboriginal and/or Torres Strait Islander descent.

Patients were ineligible to participate in the study if they had a least one of the following: a significant neurological or cognitive impairment or were unable for any reason to provide written informed consent; were pregnant; had end-stage renal failure and/or receiving renal dialysis; had a terminal malignancy requiring palliative care or limited life expectancy; required extensive support in activities of daily living; resided in an aged care or similar facility; or were incarcerated at the time of recruitment. Eligibility was reassessed throughout the study, and participants who became ineligible due to deterioration of their physical or mental health, or permanently moving outside of the geographical catchment area were withdrawn from the study.

### Recruitment

To identify potential patient participants, practice nurses (PNs) and general practitioners (GPs) provided the CMs with the names of patients with complex CD. The CMs also interrogated the practice clinical software to reveal additional patients with a diagnosis of any of the target CDs. Using the practice clinical software, the identified patients were then assessed against the inclusion and exclusion criteria and the list of eligible patients was then reviewed by the NUM, an Aboriginal nurse with strong connections to the Inala Aboriginal and Torres Strait Islander community having worked at the health service for 20 years. Based on her knowledge of the patients, the NUM made a subjective assessment of the likelihood that the eligible patients would benefit from the intensive support provided through case management and if the home environment was considered safe for home visiting by a CM; for example, patients with alcohol abuse issues, or patients living in households containing known intravenous drug users were excluded. Notation was made on the medical records of the potential participants to alert the GP to discuss the study when they next presented at the health service. At this presentation, their GP discussed the study with the potential participant and indicated the likely benefit of case management to them. If the patient agreed, they were then introduced to their allocated CM by their GP. The CM provided additional information about the study, answered any questions and invited them to participate in the study. Written informed consent was obtained from all participants prior to any data being collected for the study.

### The model of care

**Fig. 1:**
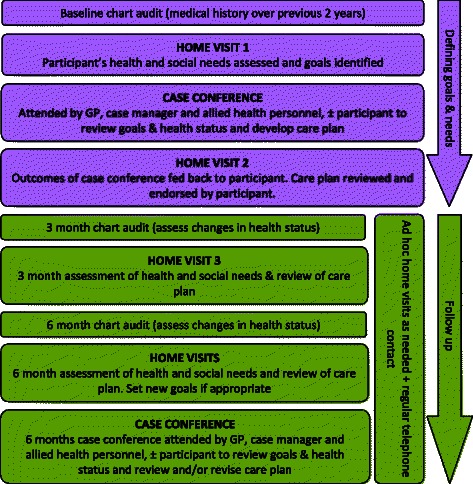
HOME Study Schema

In broad terms, the HOME Study model of care had two distinct phases. Phase one consisted of a comprehensive needs assessment that aimed to identify what each patient participant needed to be healthy and facilitated a process for them to identify their health and wellbeing goals. Phase two aimed to ensure that the health and social care systems met the identified needs of each patient participant and supported them to achieve their goals (Fig. 1).

#### Phase 1

Following receipt of informed consent, the needs assessment was completed. The needs assessment had two parts: a comprehensive audit of the participant’s medical record (chart audit) and an assessment conducted in the participant’s home of the participant physical health and social and emotional wellbeing. The chart audit included: current medications; current diagnoses; current clinical results including blood pressure (BP), diabetes control (HbA1c), body mass index (BMI), lipids, and kidney function; health service utilisation (referrals to allied health professionals, medical specialists and health or social services over the previous 12 months, and hospitalisations over the previous 24 months), and preventive health actions such as vaccinations and cancer screening. The first home visit was then undertaken, at a time that suited the participant, and an assessment conducted that covered the participant’s self-assessed health status, a depression screen using the adapted PHQ-9 measure [14], social and emotional wellbeing, lifestyle risk factors, medications (current medications, the participant’s understanding of the purpose of each medication and any problems experienced with the medications), family support, and demographic information including identification of traditional country, annual income, and highest level of schooling. Participants were encouraged by their CM to identify up to three health or lifestyle goals that were important to them, rather than those arising from previous discussions with health professionals, to focus on over the next 6 months. These might include establishing a garden, going on holiday or going back to their traditional country. This assessment also included a facilitated discussion about social, economic or health matters that would assist or impede achievement of goals. This discussion enabled the participant and their CM to collectively develop strategies for goal achievement and provided an opportunity for the CM to gain insight into each participant’s unique social, cultural and physical circumstances.

The CM synthesised the information gathered through the needs assessment, and then presented this information at a multidisciplinary case conference attended by the participant’s GP and the CM. Participants, and a family member or carer if the participant wished, were encouraged to attend prior to the case conference, the participant and their CM agreed on the key issues to be presented at case conference. The COE dietitian, social worker and/or psychologist also attended the case conference if they were involved in the participant’s care or if the CM and the participant had agreed that a particular allied health professional could assist with goal achievement and/or improving health and wellbeing. The case conference provided an opportunity for all relevant health professionals to discuss the participant’s health and social care needs and goals, and to discuss strategies to support goal achievement. The CM then re-visited the participant, where together they reviewed the outcome of the case conference and developed a care plan that clearly identified the steps and actions needed to achieve each goal, and who was responsible for each action.

#### Phase 2

Subsequent chart audits and home assessments occurred at 3 and 6 months after the initial assessment, with a follow-up case conference conducted at 6 months to review progress. In the interim, the CM concurrently fulfilled a number of roles including: facilitating progress against the goals as per the care plans, providing a point of reference for the participants when they needed assistance understanding their health care needs, advocating for participants to ensure they received the necessary health and social care services, and encouraging and empowering participants to be active members of their health care teams. It is important to note that the CM did not undertake any clinical duties as their role was one of coordination and case management, rather than the provision of domiciliary or other nursing duties.

### Outcome measures and data collection

This paper is focused on the assessment of the feasibility, acceptability and appropriateness of the model of care using data collected in the first 6 months of the study. We developed the following operational definitions of these outcomes: to be feasible, the model of care needed to be implementable; acceptable if it was able to sustain patient participants’ and health service staff engagement; and appropriate if it met the cultural, social and health care needs of the patient participants and was aligned to the aims and vision of the health service.

Assessment of the feasibility of the model of care included two elements:

- time involved in delivering the model of care, including the number of visits per participant; and
- ability to incorporate the model of care into routine practice of the health service.

Assessment of the acceptability of the model of care included five elements:

- recruitment rate and reasons participants agreed to participate or not;
- withdrawal rates and reasons people gave for withdrawing;
- evidence of no differential attrition at 3 and 6 months;
- number of participants attending case conferences at baseline and 6 months and rationales for this attendance; and
- participants’ and health professionals’ attitudes towards the model of care.

Assessment of the appropriateness of the model of care included four elements:

- changes in key clinical outcomes (BP, HbA1c, BMI and depression) and health service utilisation;
- changes in self-rated health status;
- participants’ views on how well the model of care met their cultural, social and health care needs; and
- alignment between the underlying principles of the model of care and the aims and vision of the health service.

A variety of different data sources were used to assess these outcome measures. In-depth, semi-structured interviews were conducted by the evaluator and the IRO with nine patient participants – the spouse of one patient participant also participated in one of the interviews. Fifteen COE staff were interviewed by the evaluator. Patient participants were selected to ensure representation of both genders, younger and older patient participants, higher or lower needs, and patient participants of both CMs. COE staff were selected to represent the disciplines who had the most professional contact with the CMs and HOME Study patient participants, and included four GPs, six nurses (NUM, 2 PNs, CD nurse, 2 CMs), three allied health professionals (dietitian, social worker, psychologist), and the practice manager. The HOME Study project coordinator who provided administrative support to the study was also interviewed. Study team workshops were held every 6 to 8 weeks, using a developmental evaluation approach [15]. The workshops enabled the team to better understand the minutiae of how the model of care’s implementation was adapted to meet the needs of individual patient participants and the health service. Quantitative data were collected using study specific case report forms and study specific administrative data including time logs.

#### Data analysis

With permission, participant and COE staff interviews were audio-recorded and transcribed. To ensure the faithful representation of our patient participants’ values, beliefs, knowledge and health related skills, the IRO prepared written summaries of the patient participant interviews and these were provided to the study team. Key themes relating to the feasibility, acceptability and appropriateness of the model of care were identified and discussed at the team workshops, using an iterative reflective process. The transcripts were then re-reviewed by the evaluator, an experienced qualitative researcher, to ensure that the data supported the key themes identified through this process. In a similar fashion, the evaluator prepared summaries of the COE staff interviews, and key themes relating to the aim of this initial evaluation were also identified and discussed at the team workshops. This real-time data feedback and analysis informed adaptations in the implementation of the model of care to ensure the needs of patient participants were met and improve integration with the health service. Workshop minutes were reviewed and key themes relating to the feasibility, acceptability and appropriateness of the model of care were identified and compared with the themes identified in the interviews. Where themes were divergent, further analysis of both data sets was done to resolve divergence and refine themes.

Frequencies and percentages were used to describe the baseline characteristics of our participants and to quantify the level of engagement between the participants, the CMs, the primary health care service and other parts of the health care system. Attrition and the pattern of missing values over time were evaluated using binomial generalized estimating equation (GEE) models, and mean differences of key clinical outcomes between baseline and 6 months were investigated using normal GEE models. Both GEE models used an unstructured correlation matrix and robust Huber–White sandwich variance estimators. Statistical significance for each variable measured at baseline and variable interaction with time was assessed via Wald's Type III statistic. All analyses were performed using SAS version 9.3 (SAS Institute Inc., Cary, NC, USA), and α = 0.05 defined statistical significance for all tests.

### Aboriginal and Torres Strait Islander community approval and ethical clearance

We were committed to conducting this research within the ethical framework as recommended by the National Health and Medical Research Council’s Values and Ethics – Guidelines for Ethical Conduct in Aboriginal and Torres Strait Islander Health Research [16]. The Inala Community Jury for Aboriginal and Torres Strait Islander Health Research (a group of Aboriginal and Torres Strait Islander people from the Inala community who guide all research conducted by the COE) provided community support for the study [17]. Ethical clearance was obtained from the Metro South Human Research Ethics Committee. At key points in the study, results were disseminated back to the Inala Aboriginal and Torres Strait Islander community via the Community Jury and to the COE staff at staff forums.

## Results

### Participants

**Fig. 2:**
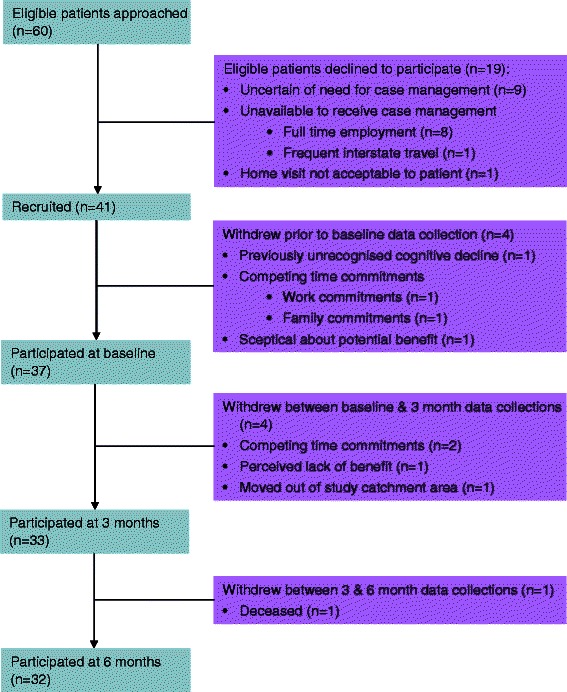
Participant Flow Diagram

We recruited 41 eligible participants and collected baseline data on 37 as four people withdrew before the initial assessment was completed; Fig. 2 presents the participant flow diagram for the first 6 months of the study.

Socio-demographic and several clinical characteristics of the participants at baseline, and the baseline characteristics of the participants remaining in the study at 3 and 6 months are presented in Table 1. All participants identified as Aboriginal, 32 (86 %) recognised a particular place as their traditional country, and 20 (54 %) had had to move away from their traditional country or family. At baseline, the mean age of participants was 59.7 years (range: 30.9–81.7 years), and 68 % were female. Overall, 59 % had an annual income less than $20,800, primary school was the highest level of schooling for 43 %, mean BMI was 35.4 kg/m^2^ (range: 23.0–63.8 kg/m^2^), and 95 % had T2D. Two participants had four CDs, five (14 %) had three, 13 (35 %) had two, and the remaining 17 (46 %) had a single CD. The majority of participants reported self-assessed health status of ‘Poor’ or ‘Fair’, with less than 10 % rating their health as ‘Very good’ or ‘Excellent’.

**Table 1:** Baseline characteristics of participants at baseline, 3 and 6 months

		Baseline (*N* = 37)		3-months (*N* = 33)		6-months (*N* = 32)	
		*n*	(%)	*n*	(%)	*n*	(%)
Gender							
	Males	12	(32 %)	11	(33 %)	11	(34 %)
	Females	25	(68 %)	22	(67 %)	21	(66 %)
Age (years)							
	<55	7	(19 %)	6	(18 %)	6	(19 %)
	55–59	9	(24 %)	7	(21 %)	6	(19 %)
	60–64	13	(35 %)	13	(39 %)	13	(41 %)
	65+	8	(22 %)	7	(21 %)	7	(22 %)
Annual income^a^							
	<$20,800	22	(59 %)	19	(58 %)	18	(56 %)
	$20,800–$31,199	6	(16 %)	6	(18 %)	6	(19 %)
	$31,200+	1	(3 %)	1	(3 %)	1	(3 %)
	Unknown	8	(22 %)	7	(21 %)	7	(22 %)
Highest level of education attained							
	Primary	16	(43 %)	15	(45 %)	15	(47 %)
	Secondary	10	(27 %)	10	(30 %)	10	(31 %)
	Post-secondary	9	(24 %)	7	(21 %)	6	(19 %)
	Unknown	2	(5 %)	1	(3 %)	1	(3 %)
Type 2 Diabetes							
	Yes	35	(95 %)	31	(94 %)	30	(94 %)
	No	2	(5 %)	2	(6 %)	2	(6 %)
Cardiovascular disease							
	Yes	14	(38 %)	13	(39 %)	13	(39 %)
	No	23	(62 %)	20	(61 %)	19	(61 %)
Respiratory conditions							
	Yes	12	(32 %)	9	(27 %)	9	(28 %)
	No	25	(68 %)	24	(73 %)	23	(72 %)
Kidney disease							
	Yes	5	(14 %)	5	(15 %)	5	(16 %)
	No	32	(86 %)	28	(85 %)	27	(84 %)
Depression or other mental illness							
	Yes	12	(32 %)	11	(33 %)	11	(34 %)
	No	25	(68 %)	22	(67 %)	21	(66 %)
Self-assessed health status							
	Poor	7	(19 %)	6	(18 %)	5	(16 %)
	Fair	14	(38 %)	12	(36 %)	12	(38 %)
	Good	8	(22 %)	8	(24 %)	8	(25 %)
	Very good	2	(5 %)	2	(6 %)	2	(6 %)
	Excellent	1	(3 %)	1	(3 %)	1	(3 %)
	No response	5	(14 %)	4	(12 %)	4	(13 %)

^a^Income categories reflect those used by the Australian Bureau of Statistics in the 2011 Census

#### Feasibility of the model of care

In the 6 to 8 months between the baseline and 6 month assessments, a total of 372 visits were made to the 37 participants (range: 2–20 visits); 346 (93 %) were conducted in the participant’s home. On average, 3.2 visits/participant were required to complete the study assessments (range: 1–6 visits/participant), 2.9 visits/participant to complete the care plan (range: 1–6 visits/participant), and 3.9 *ad hoc* visits/participant were required to implement the care plan and respond to emergent issues (range: 0–12 visits/participant). On 12 occasions, the CMs attempted a home visit but the participant was not at home, despite an appointment having been previously made and confirmed.

With respect to the time involved in delivering this model of care, the CMs spent an average of 7.0 h/participant in home visits (range: 1–16 h/participant), 2.2 h/participant travelling to appointments (range: 0.5–12.5 h/participant), 8.7 h/participant in care planning activities (range: 1.7–17.1 h/participant), 1.0 h/participant in face-to-face contact with participants at the primary health care service (range: 0–2.7 h/participant), and 30 min/participant at specialist outpatient appointments with participants (range: 0–5.5 h/participant).

Incorporation of this model of care into the routine practice of the primary health care service was facilitated through a variety of strategies. The NUM was a member of the project advisory group and the CMs’ line manager enabling the integration of the CMs into the health service’s nursing team. The CMs had full read and write access to the practice software and could review all progress notes made by the GPs in addition to updating participants’ medical records and attaching participants’ care plans themselves to ensure continuity of care. Further facilitating integration of the model of care into the health service were the myriad of “corridor conversations” the CMs had with participants’ GPs, PNs and the COE allied health professionals to ensure that all members of the primary health care team were kept fully informed about the health and social care needs of the participants. The case conferences were pivotal in ensuring integration because they provided an opportunity for the health professionals to learn from the CM, and directly from the participant if they attended, about the participants’ own priorities and concerns, and to jointly identify strategies to support the participant. Additionally, COE staff interviews included enquiry about factors that supported or constrained the integration of the model of care within the health service, and this information was fed back to the study team to enable decisions to be made to strengthen integration.

#### Acceptability of the model of care

Of the 60 eligible patients invited to take part in this study, 41 agreed (68 % recruitment rate). During recruitment, patient participants were positive about the model of care with one stating “…*would love it, it’s nice to know that someone cares [about us]*…”, another acknowledged that they needed extra care and it was “…*exactly what I need*…”, while others appreciated that the CM would be visiting them in their own homes, thus saving them from coming into the clinic. Reasons given by those who declined to participate included a belief that they were not ill enough to need the level of intensive support being offered (eight people), full time employment and not available during the day for the CM visits (eight people), home-based care not compatible with current living arrangement (one person), frequent interstate travel (one person) and the remaining person commented “…*maybe next year*…”.

A total of eight participants withdrew from the study in the first 6 months: four due to competing work or family commitments; two became ineligible to participate (one due to moving out of the study catchment and no longer attending the CoE, and the other participant was diagnosed with dementia and no longer able to provide informed consent); and two ceased to consider that involvement in the study was beneficial. One participant passed away.

Overall, withdrawal from the study was not associated with any socio-demographic or key clinical characteristics of participants. Table 1 presents the baseline profile of participants assessed at the 3- and 6-month time-points. No significant pattern of attrition was observed in any of the presented variables either by themselves or when considering interactions over time (all GEE *p*-values > 0.05).

Patient participants were always invited to attend their case conference, and the case conferences were scheduled to ensure participants could attend if they wished. Two (5 %) of 37 participants attended their baseline case conference and eight (28 %) of the 29 participants attended their 6 month case conference. The spouse of one participant also attended a 6 month case conference. Although information was not systematically collected from participants about why they attended the case conferences, feedback from the CMs indicated that participants became more involved in managing their health during the 6 months of the study, with one stating at the case conference that “*I know that I have a team behind me and that I am part of that team*”.

The interviews with patient participants and COE staff revealed that the model of care was uniformly acceptable to all interviewees. Patient participants appreciated the CM visiting them in their own homes, being interested in them and their lives, providing holistic care and removing many of the everyday stressors and worries associated with living with complex chronic diseases for them and their family members. COE staff appreciated the patient-centred case conferences and the in-depth follow-up of patients, commenting that they “…*worry less now*…” because they know that patients are not falling through gaps in the health system. The COE staff also considered that the model of care enabled them to be more proactive “…*so we weren’t always putting out bushfires, but actually doing some work underneath it*…” thereby increasing their professional satisfaction.

#### Appropriateness of the model of care

Improvements in participants’ health status suggest that the model of care met, to some extent, participants’ health and wellbeing needs. Table 2 presents key clinical indicators at baseline and 6 months. There were significant improvements in T2D control as measured by HbA1c (mean difference -0.5 %; 95 % confidence interval (CI) -1.0 %, -0.0 %; *p* = 0.05), systolic blood pressure (mean difference -13.0 mmHg; 95 % CI -18.9, -7.1; *p* < 0.001, and rates of moderate to severe depression with 62 % reporting depression at baseline reducing to 39 % at 6 months (mean difference 0.4; 95 % CI 0.2, 0.9; *p* = 0.03), but no change in self-rated health status and measured BMI (*p* = 0.57). However, information on BMI was only available from 14 patients as those with lower BMI were less likely to have their weight monitored on a regular basis and therefore more likely to have missing BMI values at 6 months.

**Table 2:** Key clinical outcome variables at baseline and six months

		Baseline	6 months	mean difference
		mean (min., max.)	mean (min., max.)	(95 % CI)
Variables assessed from medical chart audit				
	HbA1c (%)	8.0 % (6.0 %, 12.6 %)	7.6 % (5.9 %, 11.6 %)	-0.5 % (-1.0 %, -0.0 %)*
	Body Mass Index (BMI)†	39.8 (29.5, 63.9)	40.0 (31.2, 66.8)	0.1 (-0.7, 0.9)
	Blood pressure systolic (mmHg)	134.0 (101, 194)	121.7 (91, 172)	-13.0 (-18.9, -7.1)**
	Blood pressure diastolic (mmHg)	77.2 (56, 97)	74.0 (57,100)	-3.3 (-7.6, 1.0)
		n/N (%)	n/N (%)	OR (95 % CI)
Variables assessed during Home Assessments				
	Depression^a^	21/34 (62 %)	12/31 (39 %)	0.4 (0.2, 0.9)***
	Self-rated health status^b^	11/32 (34 %)	16/31 (52 %)	2.0 (0.8, 5.2)

Note: * *p* = 0.05, ** *p* < 0.001; *** *p* = 0.03† only *n* = 14 patients had both baseline and 6 months BMI values recorded and reported here; ^a^Depression assessed using adapted PHQ-9 – dichotomised as moderate to severe depression vs. otherwise; ^b^Self-rated health status dichotomised as good, very good or excellent vs. poor or fair; mean differences (95 % CI) derived from normal GEE models; and, OR (95 % CI) derived from binomial GEE models

The rate of GP consultations for acute care was, on average, 4.0 visits/participant (range: 0–20 visits/participant) between baseline and 3 months, decreasing non-significantly to an average of 3.7 visits/participant between 3 and 6 months (range: 0–23 visits/participant) (*p* = 0.56).

During the 6 months of the study, seven participants were admitted to hospital, including two participants admitted for same-day procedures. One participant was admitted twice, all others were only admitted on one occasion. In the 24 months prior to the study, 13 participants were admitted to hospital on a total of 27 occasions. Six participants had been admitted once, four had been admitted twice, three had been admitted three times and one had been admitted six times. Respiratory conditions were the most common reason for admission (13 admissions), followed by acute illnesses and cardiac conditions (6 admissions each). There were no differences in the incident rate ratios for Accident and Emergency presentations (*p* = 0.26), total admissions to hospital (*p* = 0.91) and planned admissions including same day procedures (*p* = 0.81) between the 24 months preceding the study and the 6 months of the study.

Interviews with patient participants revealed that they were positive about the model of care. The delivery of care in their own home was extremely important to participants as it increased their sense of safety and receiving comprehensive care, in addition to minimising the inconvenience and cost of having to travel to attend an appointment at the clinic or other health care facility, as these two extracts from the interviews demonstrate

*… [the CM] comes along and tells me about this program and I thought “Great, home, home”. That was the first thing I thought was “home” and I’m thinking “If [the CM] can come and see me at home that’d be wonderful for me”…* patient participant

*…the fact that I had someone coming to the home just made me feel more at ease and being able to … be more comfortable when talking about my issues…* patient participant

Patient participants reported that the model of care met their social and health care needs. They felt that their lives had been made easier because they could rely on the CMs to assist them with identifying and addressing their health and social care needs. Importantly, the CMs were able to work in a culturally appropriate manner which contributed to the development of a mutually respectful relationship between patient participants and CMs. Patient participants felt cared for, and respected, by the CMs. They also became empowered as active members of their own health care team because of the patient centred model of care – it was the patient participants’ own goals that the health care team were aiming to achieve, not goals that had been set by the health care providers. The following extracts from a patient participant and the spouse of another patient participant demonstrate the impact of the model of care on patient participants and their families

*Well it makes my life a lot easier…Like I mean it gives…us more of a life…my wife didn’t go out much because she’s stuck here looking after me…* patient participant

*I’ve been stressed, [CM] has been fantastic in being able to support me and still look after [participant’s] needs and it has had the most fantastic outcome [on] our mental and emotional health, both of us … absolutely out of sight improved and a key part of that has been [CM], at a crisis point, being able to talk with both of us…* Patient participant’s spouse

Patient participants recognised that the health professionals were working together as a team to address their health care needs, and that they themselves were key members of that team. As two participants said…

*…this is where it’s all combined in that circle like part of those cogs in the wheel. You’ve got to have them all there to be able to achieve something…* patient participant

*…so I think that’s great you know, and I do listen and I do take care and I think you know, it’s my health and they’re helping me…* patient participant

Patient participants spoke about the fundamental difference that the model of care had made to them, with one saying…

*… [I am] a different person…my whole life has just basically changed around…one of my biggest achievements [is] that I don’t need insulin anymore…* patient participant

All nine patient participants interviewed believed that the model of care had assisted them to stay healthy through the provision of practical assistance and social and emotional support. Additionally, all believed that it was a natural extension of the primary health care service, and reflected the health service’s aims and vision of providing high quality, accessible, culturally appropriate care for Aboriginal and Torres Strait Islander peoples.

Interviews with the COE staff also revealed high levels of satisfaction and a belief in the appropriateness of the model of care. There was a synergy between the model of care and the beliefs and principles underpinning primary care, in particular, coordinated and comprehensive care [18]. Similarly, synergy existed between the vision of the COE for excellence in clinical services for Aboriginal and Torres Strait Islander peoples and the principles underpinning the model of care [12]. However, the reality of the busy COE clinic meant that the care of patients with complex conditions sometimes became fragmented and reactive, rather than patient centered and proactive. Staff appreciated that the HOME Study rectified this and enabled the COE to provide excellence in clinical care through the provision of coordinated, proactive, patient centered, holistic care to the HOME Study patient participants. Attitudes of the staff are summarized by this extract from an interview with one of the GPs

*…Yes, it [HOME Study] kind of fine tunes things, the reality of complicated care within our clinic is that things always fall through the gaps. They just do, and your job is to just work as hard as you can and try to limit the gaps that things fall through. And the HOME Study kind of almost removed any gaps for those people and basically everything was done and you’d be prompted. And so their standard of care was better…it relaxes me and makes me comfortable as a doctor that we’re doing the right thing…* GP

## Discussion

Our results suggest that the HOME Study model of holistic, patient-centred outreach case management may be feasible, acceptable and appropriate for Aboriginal and Torres Strait Islander people with complex CD and for their primary health care service. The high levels of satisfaction with the model of care were verified by the participation rates and the lack of differential attrition of participants. Patient participants became more involved in their primary health care, rates of depression decreased from around two thirds to one third of participants, and significant improvements in systolic blood pressure and diabetes control were achieved. There was no change in the rates of hospitalisations, or the ratio of GP consultations for acute care compared with preventive care. The model of care, with its formal multidisciplinary case conferences and nurses dedicated to case managing the care of patients with complex health care needs, became an integral and valued strategy in the armoury of chronic disease care provided by the primary health care service.

The development of our model of care was informed by the general principles of patient centred care [19], outreach case management [10] and care coordination [20] in addition to Aboriginal and Torres Strait Islander peoples collective and holistic conceptualisations of health. We privileged each individual patient participant by ascertaining their unique requirements to attain a positive state of health, and then ensuring the health system delivered the necessary care to enable them achieve that health status. Because of our uncertainty about the how this model of care would actually be operationalised, and because we desired a level of flexibility to explore and adapt the specific manner in which the model of care would be delivered, we employed an exploratory study design. The flexibility inherent to this research design enabled us to address a variety of research questions and clarify the optimal manner in which the aforementioned principles could be operationalised in this context. However, the exploratory nature of our study, and the associated adjustments in how the model of care was implemented limit our ability to make generalisable conclusions about its efficacy [21]. We used a mixture of routinely collected clinical data and self-reported data from participants. Use of the former minimised inconvenience for patient participants, particularly as no additional tests were requested, but did result in missing data. For example, patients not receiving weight reduction care were not weighed on a regular basis when they attended the health service and therefore these data were not available for the study. Our approach to patient participant selection may have resulted in inequitable selection based on prior resource use by the patient, health service staff attentiveness or attitudes of the patient or health service staff. These factors, coupled with our requirement that the patient participant had attended the health service at least twice per year over the last 2 years may have resulted in biased selection of the more engaged patients, and non-selection of those with lower adherence, attendance or other accessibility barriers [22]. It is possible that other, less engaged patients may have benefited more from this model of care. Nevertheless, the patients that did participate in the HOME Study all had complex health and social care needs, evidenced by the high rates of depression and the low self-rated health status of the patient participants at baseline, and did benefit from the intensive supported provided through the model of care.

The CMs, based within a primary health care service, identified patient’s needs and enabled patients to identify their health and wellbeing goals. Using a social work model of case management, the CMs acted as the patient’s advocate and care coordinator to ensure that the appropriate array of health and social care services were implemented in a timely fashion [10]. People living with multi-morbidities and complex social care needs frequently experience fragmented care with limited communication and coordination across services and sectors, resulting in suboptimal care [20]. For Aboriginal and Torres Strait Islander peoples, the deleterious effects of this fragmentation are compounded by the institutional racism they so frequently experience in the health care system [23]. Our model of care aimed to address these deficits to improve health and wellbeing by combining case management with care coordination, integrated within a trusted primary health care service. Care coordination generally involves a number of players (for example, service providers, patients, and family carers) who depend upon each other to deliver disparate activities that need to be coordinated to ensure quality health care for individual patients. Knowledge of each contributor’s role, timely exchange of information, and adequate resources are required for this to occur [24]. High quality coordination is facilitated by shared goals, shared knowledge and mutual respect [25]. Integration of our model of care within a primary health care service with a shared vision of improving health outcomes for Aboriginal and Torres Strait Islander peoples facilitated achievement of these three key relational mechanisms and increased the probability that the full potential of our model of care could be realised. Further strengths of our model of care were that it was tailored to complement the existing CD care provided by the primary health care service, and that it was not constrained by discipline-specific definitions of case management or categorisation or what should, or should not be, included in the model of care.

The positive outcomes of this study have potential at the level of the individual patient, their family, the community and the primary health care system. Case management that addresses psychosocial and biomedical risk factors has provided direct benefit to individuals with CD, and to the primary health care service. Of note was the high level of satisfaction experienced by the primary health care staff who felt supported and sustained in their quest to provide high quality care to patients with complex health and social care needs. Previously, staff had felt pressured and worried because the everyday busyness of the health service prevented them providing at risk patients with the necessary level of intensive support to prevent disease exacerbations. This model of holistic, multidisciplinary patient centred care has the potential to limit the individual and population impact of chronic disease within Australia’s most vulnerable population.

This exploratory study was not without its challenges. A key challenge was the tension between the competing priorities of research and health care service delivery. This tension was ameliorated in three key ways. First, the creation of a strong team, with a shared sense of identity, purpose and understanding of each team member’s contribution to achieving the purpose increased our awareness of these tensions as they arose and our ability to manage them. Secondly, the model of care was integrated into the primary health care service which enabled effective communication about the provision of care for individual patients and the outcomes of the research. Thirdly, the exploratory nature of the study enabled the implementation of the model of care to be adapted based on feedback from the primary health care providers. In this way, the divide between researchers and service providers was decreased.

Our model of outreach case management was highly valued by the participating patients, and by the primary health care service, and further research is required to determine the sustainability of the improvements in health and wellbeing and to more fully understand the features of value of the model of care for patients and for health service staff. Identification and classification of patients most likely to achieve the greatest benefit from the intensive support could ensure appropriate resource allocation and assist with widespread introduction of the model of care.

Expansion of the model of care into other health services is required to assess what adaptations are required to implement the model in other settings and provide further evidence about the potential of the model of care. A robust evaluation, using an appropriate methodology on a larger scale, would enable assessment of the efficacy, effectiveness and cost-effectiveness of this health service intervention. Our patient-centred model of outreach case-management was resource intensive but achieved significant improvements in wellbeing and physical and mental health of our patients. Depression was a common comorbidity among our patient participants: appropriate identification and treatment may ameliorate the distress associated with living with CD, improve CD symptoms, and improve quality of life [26] thereby reducing health care costs. Additionally, a strong primary health care sector decreases health expenditure [16], and outreach case management may have the potential to decrease utilisation of secondary and tertiary health care thereby contributing to further health care savings.

This exploratory study has demonstrated that improvements can be made in the lives of Aboriginal and Torres Strait Islander peoples with complex health care needs. Continuation and expansion of this work has the potential to reshape the provision of health care that addresses the disparities in health and life expectancy in a meaningful and culturally appropriate manner.

## Conclusions

This early phase exploratory study evaluated the feasibility, acceptability and appropriateness of a home-based, case management model of patient-centred, multidisciplinary care for Aboriginal and Torres Strait Islander people with complex CD that was integrated within a primary health care service. The exploratory nature of our study preclude any definitive statements about the effectiveness of our model of care, however the high levels of satisfaction of both patients and the primary health care staff, and the improved health and wellbeing of patients are promising results. Further research is required to identify if this model of care is able to realise its potential as a culturally appropriate, effective and cost-effective mechanism to improve the quality of life and quality of care for Australia’s Aboriginal and Torres Strait Islander peoples living with CD.

### Ethics approval and consent to participate

The Inala Community Jury for Aboriginal and Torres Strait Islander Health Research (a group of Aboriginal and Torres Strait Islander people from the Inala community who guide all research conducted by the COE) provided community support for the study [17]. Ethical clearance was obtained from the Metro South Human Research Ethics Committee, HREC Reference number: HREC/12/QPAH/294.

### Consent for publication

Not applicable.

### Availability of data and materials

This manuscript reports on research conducted with Aboriginal and Torres Strait Islander peoples, a vulnerable group, and therefore require ethical oversight to be shared. Please contact Associate Professor Deborah Askew for an application to receive these data.

## Abbreviations

BMI: body mass index
BP: blood pressure
CD: chronic disease
CI: confidence interval
CKD: chronic kidney disease
CM: case manager
COE: Southern Queensland Centre of Excellence in Aboriginal and Torres Strait Islander Primary Health Care
CVD: cardiovascular disease
GEE: generalized estimating equation
GP: general practitioner
HOME Study: *H*ome-based, *O*utreach case *M*anagement of chronic disease *E*xploratory Study
IRO: Indigenous research officer
NUM: nurse unit manager
PN: practice nurse
T2D: type 2 diabetes
